# Data alchemy, from lab to insight: Transforming in vivo experiments into data science gold

**DOI:** 10.1371/journal.ppat.1012460

**Published:** 2024-08-29

**Authors:** Troy J. Kieran, Taronna R. Maines, Jessica A. Belser

**Affiliations:** Influenza Division, Centers for Disease Control and Prevention, Atlanta, Georgia, United States of America; University of Arizona, UNITED STATES OF AMERICA

Meta-analyses of laboratory-generated data have the potential to improve experimental protocols and offer meaningful understanding of complex biological processes. However, while development of genotype-to-phenotype statistical and predictive studies is growing, in vivo-generated data are generally employed for validation of genotypic models and are not often included as features in the analyses themselves, and rarely used on their own separate from genotypic data. Moreover, the difficulties of normalizing, cleaning, and transforming these data before analysis are multifaceted, in part due to challenges of aggregating sufficient data sets for practical use. Here, we highlight important considerations and best practices when translating data from viral pathogen in vivo studies for use in data science applications, notably statistical analyses, and machine learning (ML) approaches which we use as an illustrative example. Topics covered are applicable when studying multifactorial disease processes (such as viral pathogenicity and transmissibility) independent of the specific data analyses or programming language employed.

## Data transformation mastery: Bridging lab notes to analysis-ready inputs

**Fig 1:**
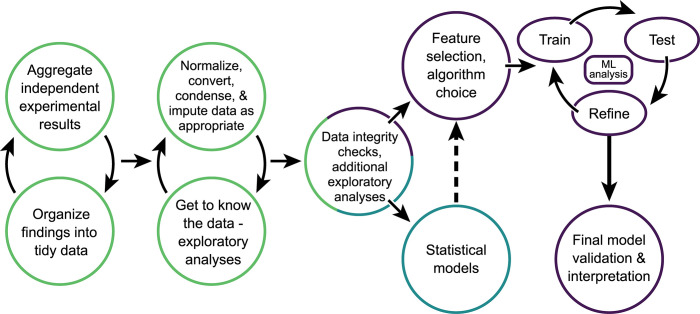
Workflow for aggregating and transforming laboratory-derived experimental results for data science use. Prior to formal analyses, diverse experimental data must be aggregated and organized into tidy data files (see Fig 2 for examples of different data types that may be sourced for this purpose). Next, additional data transformation steps will likely take place, concurrent with initial exploratory analyses to identify and refine key parameters (see Fig 2 for examples of different considerations that can modulate data transformation outcomes). Once these steps (outlined in green) are completed, hypothesis-driven research questions and model development can take place (in isolation or in tandem), such as generation of simple statistical models (outlined in blue) and ML models (outlined in purple). Establishing ML models may necessitate additional experimental considerations to be optimized prior to training/testing/refining of models. Best practices involve validating models with new, independent data, and the use of cross-validation methods to ensure accurate predictive outcomes not influenced by data noise.

When conducting an in vivo experiment, researchers will typically collect a diverse array of qualitative observations and quantitative measurements. Therefore, choosing data that are most relevant for aggregation and tidying is a crucial first step (Fig 1). Care must be taken when combining data from multiple studies to determine which data points are most consistently collected between experiments (especially if different research staff are conducting the work). This may require excluding specific parameters for analysis (like lethargy or animal activity level) which may be more vulnerable to laboratorian bias depending on the specific standardized assessment employed. To reduce experimental confounders, studies intended for aggregation should be conducted under as uniform or standard conditions as possible (with these inclusion criteria explicitly stated within the analysis) [1–3]. As any research scientist will attest, in vivo-generated data is highly heterogeneous, particularly when using outbred species. Variability may be present in baseline (pre-inoculation) animal age, weight, temperature, activity level, blood chemistry, and innate immune response parameters, among others. Inoculation (e.g., infectious dose) and post-inoculation (e.g., specimen collection) variability can also be present. As most studies assessing viral pathogenicity report changes relative to baseline, normalizing raw data to reflect a linear or percentage-based deviation from baseline will typically yield aggregate data with less standard error and greater uniformity, and represents a best practice in the field [4]. Normalization can typically occur before or after aggregation.

**Fig 2:**
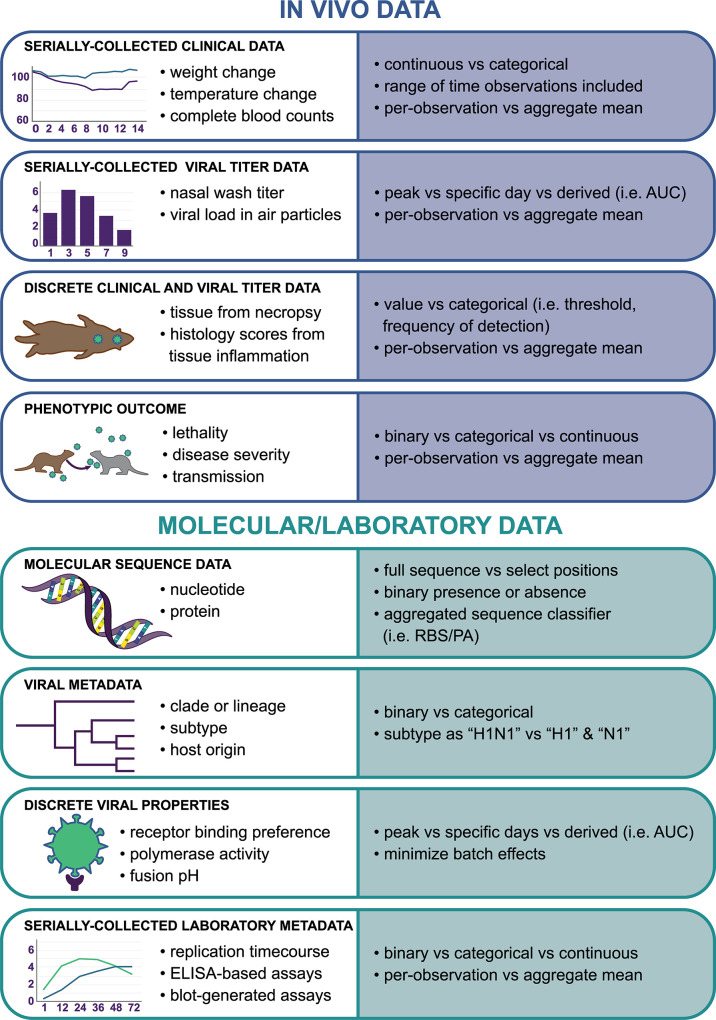
Considerations when aggregating and tidying in vivo and molecular/laboratory data. In vivo-generated data can encapsulate a wide range of serially collected and/or discrete (stand-alone) specimens and observations, and experimental outcomes. Results from in vivo experimentation are frequently contextualized with a diversity of pathogen sequence-based information and laboratory-based assays. Examples of data types within these groupings are shown on the left-hand side of this figure. Depending on the data type, there are a range of options available for distilling complex laboratory-based readouts into discrete values which are necessary for many data science applications; these decisions can meaningfully impact the conclusions drawn from the work. Examples of how complex data can be tidied for this purpose for each data type are shown on the right-hand side of this figure. AUC, area under the curve; RBS, predicted receptor binding preference; PA, predicted polmerase activity. Data types and analysis considerations are representative only and do not encapsulate all potential parameters employed in data science applications employing in vivo data. Image generated entirely by CDC illustrators by hand.

It is frequently desirable to contextualize in vivo-derived outcomes with genotypic data [5–8]; however, these data must be similarly curated before further analysis, with reliable consensus sequence data available for aggregation and use (Fig 2). Will full-length genetic sequences be assessed, or will specific molecular residues that are known to affect the tested variable be sufficient [9]? Molecular residues are often compensatory in nature; will researchers build new data set columns with anticipated phenotypic outcomes from constellations of specific amino acids at key positions (like predicted receptor binding preference or length of an accessory protein)? If laboratory-generated data will be included, have researchers ensured reproducibility of aggregated experiments performed over time [10], with oversight for potential dual-use research of concern? Considering the scope of information that can be obtained from in vivo, in vitro, and molecular analyses, selecting input data for subsequent processing represents a challenging endeavor.

## Distilling complexity: Turning serial data into single values

In vivo pathogen experiments typically yield both serially collected and discrete viral titer and clinical data (Fig 2). Serially collected data (especially linked measurements over time) can provide valuable information about pathogen biology, but normalizing, converting, and condensing serially collected data into discrete values, often employed in many standard statistical and predictive applications, can represent a substantial challenge, and a choice that may greatly influence the resulting analyses (Fig 1). In example, serially collected infectious virus titers from the nasal passages of a virus-inoculated animal can be reported as a mean peak titer, a discrete titer representing one sampling time point, an area-under-the-curve summary measure, a measure of infection progression between multiple time points, among others [11,12]. Distilling these serial data into discrete values may necessitate a great deal of exploratory analyses to ascertain data structure and variable relationships, while concurrently ensuring that associations identified in the data are biologically relevant and in alignment with known behaviors of the model pathogen(s) tested.

Furthermore, it may be prudent to consider if selected numerical summary parameters, with varying degrees of biological effect which can generate spurious noise in a model, should be transformed to other data types prior to analysis (Fig 2). Is it more sensible to convert maximum weight loss from a continuous to a categorical variable, and if so, how many weight loss categories are appropriate? Is it more appropriate to report viral detection in a tissue over (or under) a predetermined cutoff rather than using a quantitative titer measurement? Which criteria determine whether a binary report of a phenotypic outcome is optimal, and if so, which one? These decisions may result in greater statistical strength and/or predictive outcomes, but often cannot or will not be made until exploratory analyses are completed. However, researchers should never eliminate raw data, but rather add to the dataset these modifications and transformations with applicable accompanying code to reproduce them.

## Unveiling data: Scales/model choice driven by data

It is crucial to decide if in vivo source data are best represented at the level of individual animals or as a mean/median of multiple animals (inoculated with the same virus, treated with the same agent, etc.); this may not be finalized until late in the study (Fig 1). A smaller sample size may result from aggregating in vivo data at the level of viral inoculation or treatment condition but may increase consistency between like groups with fewer outliers. While it is reasonable to link data points collected from the same animal over time, it might not be possible to link data collected from multiple animals across the same observation for analyses (like ML) which require one row of data per observation (e.g., eschewing pairing per-animal necropsy data with serially collected data from different animals, so not to imply a linkage in viral titers obtained between different individual animals). This may necessitate mixed rows in the data set encapsulating in vivo-derived data at both the per-animal and per-virus level, alongside molecular data, and any additional in vitro-based parameters (which may exhibit some variability across scales).

Concurrent with these analyses, elucidating the underlying relationships present in data across scales will ultimately contribute towards improved analysis approaches. Running statistical correlations, linear regression models, and other assessments would inform if the relationships meant to be explored are linear or not [11,13–15]. For ML applications, understanding if predicted outcomes should be categorical classification, numerical regression, or clustering, supervised or unsupervised, can represent a crucial decision that will greatly impact the suitability of ML algorithm(s) employed and results obtained [16,17]. On a positive note, it is likely that these formative analyses on source data will provide valuable information towards subsequent understanding of disease processes.

## Unlocking the code: Seamlessly integrating the data science recipe

As exploratory results are obtained, it is critical to apply a strong mix of domain expertise (and common sense) when determining biological relevance for downstream analyses like multivariate statistical evaluates and/or feature selection in ML [5]. Different data types obtained during laboratory experimentation may govern the choice of ML algorithm(s) employed [16]. Determining which features, or variables, to use in ML is also dependent on data availability. In vivo studies often have features with missing data (such as resulting from an animal reaching an unscheduled humane endpoint, or serially collected weight/temperature observations recorded on different schedules post-inoculation). Determining whether such a feature should be dropped or have values imputed usually comes down to the amount of missingness and how biologically important such a feature may be. Many methods for data imputation exist with their own considerations [17].

## From raw input to reliable conclusions: Interpreting and validating results

Regardless of the analyses conducted, it is quite possible that in vivo source data will not always be associated with high statistical correlations and/or high-performance metrics using ML algorithms. This should not be considered a failure. Studies conducted in vivo (including but not limited to studying pathogen pathogenicity, tropism, and transmissibility) are conducted specifically because molecular, in vitro, and/or ML algorithms are insufficient to fully predict these multifactorial outcomes. There is great utility in understanding the relative contribution of specific features from in vivo experimentation towards disease outcomes, even if the highest statistical correlation or performance metric itself may not be as striking, or the confidence intervals of an association are higher than desired [10,11,18,19].

For ML studies, it is crucial to confirm any high-performance metrics obtained, ideally with externally generated independent testing data, to ensure model overfitting is not taking place (Fig 1) [13,16]. However, this can pose a substantial challenge when employing in vivo data in training data sets, due to a paucity of publicly available data sets for testing. The potential for substantial heterogeneity across different laboratories performing similar research, and the likelihood that selected features required for model use are not available in other published studies, must be considered. Researchers can employ techniques like cross-validation, or combing internal and external data together in both the training/testing data sets to improve model robustness. However, care must be taken for practicality and appropriateness. It is likely that researchers will need to comb the literature to create from scratch suitable data sets for evaluation and validation purposes [5,19], despite this being a time-intensive effort.

## Spreading the data science wealth: An alchemist’s guide

The diversity of data science analyses possible with data aggregated from in vivo experimentation likely exceeds what any one research group can identify on their own. Publicly releasing aggregated data sets for use by other researchers represents a best practice for data sharing in general, and supports the 3 R’s of animal research by seeking to glean additional insight from preexisting data without employing additional animals. Preparing these data for external dissemination can be a challenge, but making sure the data is compiled in a digital (i.e., CSV) tidy format (i.e., with one observation per row and one variable per column), with associated metadata and code for how the data was collected, complied, and modified, and deposited in a public database is important for transparency, reproducibility, and scientific advancement. Additionally, data sets can be described in detail in companion peer-reviewed journals that publish these Data Notes or Data Descriptors, which can be cross-referenced and cited to primary manuscripts, encouraging reuse, with complete information about the creation, benefits, and limitations of the data (as an example see [20]).

## Conclusions

In vivo studies provide critical information that cannot be obtain from in vitro and/or molecular analyses alone. However, retrospective analyses employing in vivo-generated data are rare [5,11,18], with few viral pathogen studies incorporating in vivo data in ML algorithms [13,19]. While we focus on ML as an illustrative example, these principles apply to other statistical methodologies not discussed here. As discussed throughout this article, these analysis workflows involve numerous decisions that necessitate the input and domain expertise of both data scientists and research staff. The most rigorous and valuable studies will ultimately result from close collaboration between groups who perform in vivo research and groups who perform meta-analyses of this work. While it can be time consuming, the benefits of data aggregation and sharing can be transformative, and increased efforts by researchers to generate and share these data resources are warranted.

## Disclaimer

The findings and conclusions are those of the authors and do not necessarily reflect the official position of the Agency for Toxic Substances and Disease Registry (ATSDR)/the Centers for Disease Control and Prevention (CDC).
